# Development of immunotherapy for high-grade gliomas: Overcoming the immunosuppressive tumor microenvironment

**DOI:** 10.3389/fmed.2022.966458

**Published:** 2022-09-14

**Authors:** Andrea Franson, Brandon L. McClellan, Maria Luisa Varela, Andrea Comba, Mohammad Faisal Syed, Kaushik Banerjee, Ziwen Zhu, Nazareno Gonzalez, Marianela Candolfi, Pedro Lowenstein, Maria Graciela Castro

**Affiliations:** ^1^Division of Pediatric Hematology/Oncology, Department of Pediatrics, University of Michigan Medical School, Ann Arbor, MI, United States; ^2^Department of Neurosurgery, University of Michigan Medical School, Ann Arbor, MI, United States; ^3^Department of Cell and Developmental Biology, University of Michigan Medical School, Ann Arbor, MI, United States; ^4^Immunology Graduate Program, University of Michigan Medical School, Ann Arbor, MI, United States; ^5^Instituto de Investigaciones Biomédicas (INBIOMED, UBA-CONICET), Facultad de Medicina, Universidad de Buenos Aires, Buenos Aires, Argentina; ^6^Department of Biomedical Engineering, University of Michigan Medical School, Ann Arbor, MI, United States; ^7^Biosciences Initiative in Brain Cancer, Biointerface Institute, University of Michigan, Ann Arbor, MI, United States

**Keywords:** gliomas, immunotherapy, tumor microenvironment, gene therapy, immune suppression

## Abstract

The preclinical and clinical development of novel immunotherapies for the treatment of central nervous system (CNS) tumors is advancing at a rapid pace. High-grade gliomas (HGG) are aggressive tumors with poor prognoses in both adult and pediatric patients, and innovative and effective therapies are greatly needed. The use of cytotoxic chemotherapies has marginally improved survival in some HGG patient populations. Although several challenges exist for the successful development of immunotherapies for CNS tumors, recent insights into the genetic alterations that define the pathogenesis of HGG and their direct effects on the tumor microenvironment (TME) may allow for a more refined and targeted therapeutic approach. This review will focus on the TME in HGG, the genetic drivers frequently found in these tumors and their effect on the TME, the development of immunotherapy for HGG, and the practical challenges in clinical trials employing immunotherapy for HGG. Herein, we will discuss broadly the TME and immunotherapy development in HGG, with a specific focus on glioblastoma multiforme (GBM) as well as additional discussion in the context of the pediatric HGG diagnoses of diffuse midline glioma (DMG) and diffuse hemispheric glioma (DHG).

## Introduction

Despite substantial advances in the treatment of several types of cancer in both adult and pediatric patients in recent decades, the standard treatment of high-grade glioma (HGG) remains unchanged with up-front surgical resection followed by radiation therapy (+/- concurrent chemotherapy) and outcomes remain very poor (1, 2). The successful development of immunotherapies for the treatment of select hematologic malignancies and solid tumors with CAR T cells and immune checkpoint blockade, for example, has led to rapid-pace growth in the field of anti-cancer immunotherapy. The development immunotherapies in the treatment of many solid tumors, HGG included, has been limited by several factors, most notably the anti-inflammatory tumor microenvironment (TME) that is frequently found in the peri- and intratumoral immune cellular milieu (3, 4). This pro-tumor and anti-inflammatory TME is influenced by many factors, including the specific tumor subtype, the genetic aberrations found within the tumor, and the host immune system. Additional considerations in immunotherapy development in the treatment of tumors of the brain/spine include the ability of therapy to cross the blood-brain barrier (BBB) or the use of a local delivery method to bypass the BBB (5). Immunotherapies currently in development are mechanistically diverse, and each therapeutic approach involves complexities in preclinical and clinical study design that must be carefully considered for rigorous data to result. As more of these therapies are transitioned into clinical trials in pediatric and adult patients, a deep understanding of the expected local and systemic immunologic effects is essential to an appropriate trial design and incorporation of the appropriate bio-correlative studies. Successful clinical development of immunotherapies for HGG is likely to require a multi-institutional, consortia-based approach, with collaborations between academia and pharmaceutical companies. Here, we review in depth the TME in HGG, the various drivers of the TME in HGG, a current review of various immunotherapeutic approaches, and some of the challenges to successful immunotherapy development.

## Tumor microenvironment in high-grade glioma

### Myeloid derived suppressor cells (MDSCs)

High grade gliomas are heterogeneous tumors, with a complex composition of both malignant and non-malignant cells. The majority of HGG-infiltrating cells are macrophages, microglia, and dendritic cells (DCs), with non-tumor cells make up ~50% of HGG total tumor mass (6). The tumor microenvironment (TME) is composed primarily of myeloid cells, including DCs, neutrophils, tumor-associated macrophages (TAMs), and myeloid-derived suppressor cells (MDSCs) (7).

MDSCs are found extensively in the HGG TME (8, 9), and they are an heterogeneous population of immature myeloid cells (10). MDSCs are dominant intratumoral immunosuppressive cells and have been found to directly interfere with the efficacy of immunotherapy (11). Two distinct subpopulations of MDSCs have been described, polymorphonuclear (PMN-MDSC) and monocytic cells (M-MDSC), with PMN-MDSCs being the more abundant in several tumors, including glioma (12–14). Patients with HGG have elevated levels of circulating MDSCs, with high levels of PMN-MDSCs that continue to expand during glioma progression and negatively correlate with patient survival (15, 16).

MDSCs block an anti-tumor response by the immune system by suppressing effector T cells and inducing Tregs (8, 17). MDSCs can produce arginase (Arg I), reducing the amount of L-arginine available for T cells and necessary for their normal function. MDSCs can also secrete nitric oxide (NO) leading to the production of reactive oxygen species (ROS), which themselves are capable of inducing T cell suppression. Additionally, MDSCs can express PD-L1, resulting in the upregulation of PD-1/PD-L1-mediated immunosuppression (18, 19). The production of Arg I and NO is the primary immunosuppressive mechanism of M-MDSCs, whereas the production of ROS is the primary mechanism by which PMN-MDSCs act on effector T cells (20). Gliomas produces cytokines that can recruit MDSCs (i.e. CCL2, CXCL8, SDF-1, CXCL2), as well as cytokines that induce MDSCs expansion (i.e., IL-6, PGE2, IL-10, GM-CSF) (21). Additionally, the TME of HGG is generally hypoxic, leading to upregulation of vascular endothelial growth factor (VEGF) and hypoxia-inducible factor-1 alpha (HIF1a) and therefore enhanced migration of MDSCs to the tumor (22). The STAT3 pathway is often constitutively active in HGGs, and STAT3 activation induces the secretion of immunosuppressive cytokines, suppressing T cell expansion and promoting Tregs recruitment, and promotes tumor angiogenesis (23, 24).

Temozolomide (TMZ) is the most used cytotoxic chemotherapy for the treatment of HGG. Treatment with TMZ in preclinical models leads to an increased production of HIF-1a and VEGF that stimulates the expansion of MDSCs (25). Possible therapeutic approaches to target the immunosuppressive activity of MDSCs include the inhibition of COX2 to reduce MDSCs recruitment and reduce Arg I expression (26) and blocking CSF-1, CXCL2, or CXCL12 to inhibit trafficking of MDSCs to the tumor (20).

### Glioma-associated macrophages and microglia (GAMs)

The immune suppressive TME is a hallmark of GBM (27). The glioma TME includes several non-neoplastic cell populations, and within them, the glioma-associated macrophages and microglia cells (GAMs) make up more than 30% of tumor mass (28). GAMs contribute to the immunosuppressive TME milieu, and as a result, they represent an attractive therapeutic target to overcome resistance to anti-tumor therapy (29). Microglia and macrophages have different ontological origin and differentiating these cell types within the tumor microenvironment is difficult (30). Microglia cells are the resident macrophages of the brain, originated from progenitor cells in the yolk sac that migrate to the brain during embryo development (31, 32). In contrast, macrophages are myeloid immune cells derived from circulating monocytes that infiltrate the tumor (33). Although, microglia/macrophages participate in the normal brain immune surveillance and tissue homeostasis, their phagocytic and pro-inflammatory role is altered during the progression of gliomas. Instead, they promote tumor proliferation, increase tumor cell migration, and support immune suppression (34).

Several paracrine signals secreted by glioma cells lead to the recruitment of GAMs to the tumor and drive them into a pro-tumorigenic state (34). As a result, GAMs represent potential targets to reprogram the tumor microenvironment and impair tumor malignancy.

Recent studies indicate the role of macrophages in inducing glioma mesenchymal-like states through stimulation of Oncostatin-M (OSM) and its receptor OSMR, resulting in STAT3 activation (35). Additionally, another recent study shows that COL1A1 inhibition in glioma cells decreased CD68+ and IBA1+ macrophages/microglia within the tumor, decreased mesenchymal transformation, and inhibited tumor growth (36).

Glioma-derived cytokines such as the colony stimulating factor 1 and 2 (CSF1/2) stimulate infiltration of GAMs and promote an M2 anti-inflammatory, pro-tumoral phenotype (37). In this context, blocking its receptor, CSF1R, led to impaired recruitment of GAMs and reduced tumor invasion (38). Inhibitors such as RG7155, BLZ945 and, PLX339 have been used to block CSFR1 in clinical trials, however results are inconclusive and require further interrogation (39). It has been shown that the inhibition of CXCL12/CXCR4 signaling pathway, in combination with radiotherapy, decreased myeloid cells' infiltration; delayed tumor progression and ultimately led to anti-glioma immunological memory (40).

Recently, it has been demonstrated that CD47 overexpression in glioma cells helps tumor cells to escape phagocytosis and correlates with decreased overall survival (41). CD47 is a ligand of the SIRPα receptor expressed in macrophages. Therapeutic inhibition of CD47 using specific antibodies in preclinical orthotopic models of solid tumors, including GBM, showed a decrease in tumor growth increasing animal survival (42). A latest clinical trial using 5F9, a CD47 inhibitor, in combination with other anticancer therapy showed promising results in solid tumors (43). Moreover, the interaction of overexpressed sialic acid within glioma cells and the SIGLEC (sialic acid-binding immunoglobulin-like lectin) receptors in macrophages was identified as a negative regulatory mechanism of phagocytosis. Antibody ablation and genetic inhibition of one of the SIGLEC family members (SIGLEC15) increased antitumor immune response and reduced tumor progression in preclinical mouse glioma models (44).

The reprograming of GAMs appears to be a promising approach to inhibit glioma progression. However, due to the inherent heterogeneity of HGGs, it is plausible that single modulation of GAMs activity and infiltration will not be sufficient by itself. Thus, integrative approaches to target GAMs in combination with other immunotherapies such as CAR T cells, checkpoint inhibitors, or vaccine treatments needs to be further evaluated.

### T cell infiltration and dysfunction in high-grade glioma

Poor anti-tumor T cell response is a hallmark of HGG (45). The most notable causes for this hampered T cell response are low T cell infiltration and TME-induced T cell dysfunction (46). Low numbers of antigen presenting cells (APCs), the presence of the blood-brain barrier (BBB), and the immunosuppressive microenvironment all contribute to the poor anti-tumor T cell activity (47, 48). Under typical inflammatory conditions, APCs such as DCs take up antigens, migrate to the draining lymph nodes, and present the antigens and costimulatory signals to T cells. The combination of antigen and costimulatory signals with T cell receptors (TCRs) that recognize and bind to the antigen results in T cell activation and proliferation. These T cells migrate to the original location and activate targeting of the antigen expressing cell (49). In the case of glioma-mediated brain inflammation; the paucity of APCs present in the brain parenchyma, and the immunosuppressive TME (50, 51) limit the ability of APCs to take up tumor antigens, traffic to the draining lymph nodes and stimulate efficient activation of T cells (52). Cytokines in the HGG TME, such as TGF-B and IL-10, also inhibit DCs activation and drive DCs polarization to an anti-inflammatory phenotype with low costimulatory molecule expression (51, 53). Thus, as the tumor develops and produces cancer-specific antigens, the APCs in the TME have a limited ability to activate and recruit T cells due to their limited number and the immunosuppressive environment.

Although the location of HGG contributes to poor anti-tumor immunity, the immunosuppressive TME also plays a major role by activating several immunoregulatory mechanisms to induce T cell dysfunction. Along with glioma cells inducing T cell apoptosis through the expression of FasL (54), the primary mechanisms of T cell dysfunction are through the induction of T cell exhaustion and T cell tolerance. T cell exhaustion is a state of activated yet hyporesponsive, or non-responsive, T cells resulting from long-term antigen exposure under immunosuppressive conditions (55, 56). Under normal, acute inflammatory conditions, these immune checkpoints are beneficial, in that, they dampen the immune response of T cells to prevent damage to healthy tissue within the host (55). In the case of chronic infections and cancer, such as HGG, these mechanisms of immune dampening hinder the pro-inflammatory, anti-tumor immune response. Exhausted T cells are marked by “immune checkpoint” receptors including PD-1, CTLA-4, TIM3, LAG3, TIGIT, and several other newly-emerging molecules, and the binding of these receptors to their cognate ligand promotes the reduced T cell functionality (57). In general, glioma cells have been shown to promote exhaustion directly by expressing the checkpoint receptor ligands PD-L1, Galectin-9, B7-H4, B7-H3, CD155, HVEM, PD-L2, CEACAM-1, but there are differences in which ligands are expressed depending which GBM cell line is considered (58). This variation is attributed to differences in GBM cell mutational burdens (56, 59–61). PD-L1 expression, which is perhaps the most researched immune checkpoint receptor ligand, is reduced on glioma cells harboring a mutation of isocitrate dehydrogenase gene 1 (IDH1) and is increased after loss of phosphatase and tensin homolog (PTEN) (60, 62, 63). These findings suggest the need to identify additional links between HGG cell mutational burden and immune-suppressive ligands. Notably, the features and levels of T cell exhaustion correlates with the aggressiveness of the GBM (56, 58).

Like T cell exhaustion, T cell tolerance is a state of restricted T cell responses that are normally activated to prevent injurious T cell responses (64). GBM cells alter the TME to induce T cell tolerance through immunosuppressive myeloid cells (discussed above) and T regulatory cells (Tregs) (52). Tregs are a subset of CD4+ T cells, which are primarily identified by their expression of the transcription factor Foxp3. The trafficking of Tregs to the TME is mediated, at least in part, by Treg chemoattraction to GBM-derived chemokines CCL2 and CCL22 (65, 66). The GBM-secreted indoleamine 2,3-dioxygenase 1 (IDO) also supports Tregs accumulation as high IDO levels in the GBM TME are positively associated with Treg accumulation and negatively impact survival (67). Tregs limit the antitumor immune response through multiple mechanisms including: secreting the anti-inflammatory cytokines IL-10 and TGF-B, sequestration of IL-2, the cytokine necessary for T cell proliferation, converting ATP to AMP in the immunosuppressive adenosinergic pathway, inhibition of DCs by CTLA-4 expression, FasL-mediated T cell apoptosis, and expressing T cell exhaustion promoting ligands such as PD-L1 (68, 69).

## Genetic drivers of high-grade glioma and tumor microenvironment

### IDH mutations and effects on the TME

Isocitrate dehydrogenase (IDH) is an enzyme involved in cellular metabolism and oxidative stress responses, catalyzes the conversion of isocitrate to α-ketoglutarate (αKG). Mutations in IDH (mIDH) are common genetic lesions in HGGs (70). About 90% of IDH1 mutations occur at codon 132 of exon 4, resulting in a single amino acid change from arginine to histidine (R132H). Mutation of active site residue R132 converts αKG to 2-hydroxyglutaric acid (2-HG) (71, 72). Excessive 2-HG leads to DNA hypermethylation by inhibiting the methylcytosine dioxygenase TET2 and promotes histone hypermethylation by competitively inhibiting αKG-dependent Jumonji-C histones demethylases (73, 74). This hypermethylation results in epigenetic reprogramming of the transcriptome within mIDH1 glioma cells (75).

Multiple studies have shown that IDH mutational status affects the immunological landscape of the TME. Mutations in IDH in HGG have been associated with reduced expression of programmed death ligand 1 (PD-L1) is (due to hypermethylation of the CD274 promoter) expression and less infiltration of CD8+ T cells (62, 63, 76, 77). This reduction in T cell infiltration was a result of decreased expression of adhesion molecule ICAM1 and chemo-attractants CXCL9 and CXCL10, which together mediate the recruitment of T cells from circulation into the TME (78). The decreased T cell infiltration and consequent decreased IFN-γ levels in the TME may also play a role in reducing PD-L1 expression (77). There is also evidence that mIDH is also involved in immune evasion mechanisms through the reduction of STAT1 levels and inhibition of CD8+ T cells accumulation (77). A low proportion of CD8+ T cells is also seen in the presence of reduced chemokine expression (79). A recent study revealed a significant reduction in CD4+ T cells and Foxp3+ Tregs in mIDH glioma (80). Tregs were prevalent in tumors of astrocytic lineage, predominantly, high grade gliomas (81). In addition, mIDH1 glioma cells have been observed to have lower expression of NKG2D, a receptor that activates NK cells and CD8+ T cells and mediates cytotoxic effects on target cells. Thus, decreased NKG2D expression results in mIDH1 cells evading NK immune surveillance (82). Another study demonstrated that 2-HG can also cause elevated levels of p-NF-κB, which promote the expression of the chemokine CX3CL1 and then leads to the recruitment of more NK cells into the TME (83). Furthermore, mIDH gliomas have been shown to exhibit higher methylation of the MHC-I HLA, which reduces MHC-I expression levels in these tumors (84). MHC-I is important for NK cell-mediated lysis, as lack of HLA class I molecules or their downregulation is accompanied by upregulation of ligands that activate NK receptor recognition, thereby promoting NK cell-mediated lysis (85).

It has been reported that immune cells infiltrating mIDH gliomas have features distinguishing them from immune cells infiltrating wtIDH tumors. Interestingly, the major types of TAMs differed between mIDH and wtIDH gliomas, with mIDH TAMs consisting mainly of microglia and wtIDH gliomas mainly associated with monocyte-derived macrophage (86, 87). However, microglia also appear to be present in wtIDH glioma and are more activated than those found in mIDH glioma based on CD14 and CD64 expression (86). Friedrich *et al*. show that IDH-mutant gliomas educate their infiltrating macrophages toward an immunosuppressive phenotype through regulation of tryptophan metabolism (88). Nevertheless, this mutant IDH1 mouse glioma model does not harbor ATRX and TP53 inactivating mutations, thus it does not take into account the genetic context encountered in diffuse astrocytic lower grade gliomas (70). The presence of MDSCs in the HGG TME has been linked to reduced numbers of tumor-infiltrating T cells (89). Notably, recruited monocytic MDSCs may in turn differentiate into TAMs in glioma (90). Interestingly, the glioma TME-generated CCL2 mediates the recruitment of inhibitory CCR2+ monocyte MDSCs and Tregs (91), while mIDH glioma has a reduced CCL2 expression (76). When compared with wtIDH gliomas, mIDH tumors have also been shown to have fewer infiltrating neutrophils (62, 76, 84). In our recent study, we showed that granulocytes in mIDH tumors did not exhibit immunosuppressive properties compared to infiltrating granulocytes within wtIDH tumors (9). In these mice, the presence of mIDH1 reprogrammed the transcriptome of tumor cells, affecting not only immune cell infiltration but also granulocyte differentiation in the bone marrow (9). Furthermore, immunostimulatory gene therapy showed higher efficacy in mIDH1 glioma than in wtIDH1 glioma tumor-bearing mice, and this effect depended on G-CSF secreted by mIDH1 glioma stem-like cells (9).

### Cytokines as possible driver of tumor immune escape

Cytokines are multi-faceted molecules in the TME that regulate neo-angiogenesis, proliferation, invasion, and immune cell infiltration. Tumor-associated cytokine dysregulation causes the immune system to fail to detect tumor cells, suppressing effective cell-mediated immunity (92). The complex cytokine network in the TME supports HGG growth by allowing crosstalk among normal brain cells, tumor cells, and immune cells (93). HGGs expresses a variety of immune-suppressive cytokines, including TGF-β, IL-10, IL-4, IL-6, and IL-13, all of which impede the anti-glioma immune response directly or indirectly (94–98). IL-33 has been demonstrated to promote glioma growth, decrease overall survival, and orchestrate the GBM microenvironment to overcome immunotherapy resistance (99). IL-6 and CSF-1 are pro-inflammatory cytokines that cause an immunosuppressive environment in GBM by suppressing T cell functions (100, 101).

Despite recent advances in cancer immunotherapy, HGG remains highly resistant to a variety of immunotherapies due, in part, to a TME that inhibits the anti-tumor immune response. The GBM microenvironment elicits T cell exhaustion, characterized by upregulation of multiple immune checkpoints and transcriptional signatures. However, T cell exhaustion cannot be reversed with immune checkpoint blockade alone, emphasizing the urgent need to identify other underlying mechanisms of glioma-induced exhaustion to develop effective immunotherapies targeting HGG (56). An additional target of immunotherapy could be TAMs, which are recruited to the glioma TME and release anti-inflammatory cytokines, such as transforming growth factor β (TGF-β) and IL-10, and pro-inflammatory cytokines including TNF-α, IL1-β and CXCL10 (28, 102). As discussed above, macrophage-derived Oncostatin-M (OSM), a member of the IL-6 family of cytokines, triggers a mesenchymal-like GBM state with enhanced cytotoxicity of T cells, altering the TME (35).

Recently, a subset of IL-10-releasing HMOX1^+^ myeloid cells spatially localized to mesenchymal-like tumor regions were found to drive T cell exhaustion and trigger an immunosuppressive TME (103). Additionally, these TAMs have a distinct effect throughout the entire tumor mass, where blood-derived macrophages that predominate in the center of the tumor exert immunosuppressive effects (28). In contrast, microglia derived CCL5 can augment low-grade glioma growth (104). Glioma-derived cytokines are the primary drivers of MDSC expansion in the TME. MDSCs also exert their immunosuppressive effects by differentiating into TAMs within the tumor by producing anti-inflammatory cytokines, reactive oxygen species (ROS), and nitric oxide and interfere with anti-glioma immunotherapy (8, 9, 105). Furthermore, constitutive STAT3 signaling has been linked with increased MDSCs and TAMs within the TME (106). An inhibitor of STAT3, WP1066, has been recently shown to have anti-tumor activity in histone H3 G34R-mutant HGG, a subtype of HGG most commonly occurring in teenage and young adult patients (107), and is currently being evaluated in clinical trials (NCT01904123 and NCT04334863).

An interesting strategy utilizing convection-enhanced delivery (CED) to infuse GBM cells with a recombinant form of IL-13 fused to Pseudomonas Exotoxin A (IL13-PE38QQR) was evaluated in a phase 1/2 clinical trials for the treatment of patients with recurrent malignant gliomas (NCT00041587). This study demonstrated that local administration of IL13-PE38QQR is safe (108, 109). Recently, the antibody-cytokine fusion molecule L19-TNF, when administered after systemic treatment, transports immune-stimulatory cytokines directly to tumors to evoke immune responses (110). The antibody-cytokine fusion protein is injected intravenously and accumulates in tumors, demonstrating promising effects in mice models. A phase 1/2 clinical trial to evaluate the safety and early efficacy of L19-TNF was completed in patients with metastatic solid tumors and safety objectives were achieved (111). Currently, a phase 1/2 trial with L19-TNF in patients with HGG at first relapse is ongoing (NCT03779230).

### Pediatric high-grade glioma

Diffuse midline glioma (DMG) is a subtype of pediatric HGG (pHGG) and is one of the most lethal pediatric CNS tumors (1, 2). DMG arises in midline structures, such as the pons, thalamus, and spinal cord, and the median overall survival (OS) in patients with DMG remains at just 9 to 11 months despite much ongoing clinical and translational research in this area (1, 2). The understanding of the genetic and epigenetic landscape of DMGs has deepened substantially in the last decade, from the initial description of recurrent somatic mutations in the histone H3 genes (112–114) to the recent expanding cataloging of the frequently altered pathways in pHGG (1). The epigenomic rewiring that occurs as a result of histone mutations lead to significant alteration in the post-translational histone modifications that then drive differential transcriptional output (114, 115). Additionally, there is a growing list of recurrent mutations found in DMGs, such as *TP53, PDGFRA, PTEN, PIKC3A*, and *ATRX*, as well as the use of methylation profiling to prognosticate based on tumor sub-grouping (116–120). Multiple ongoing preclinical and clinical trial efforts are underway to determine if targeting these altered genes using targeted therapy leads to anti-tumor activity as monotherapy as well as in a combination approach with agents such as TKIs, PI3K inhibitors, and HDAC inhibitors, among others (121) (NCT03739372).

Another predominately pediatric subtype of HGG is histone H3 G34-mutant diffuse hemispheric glioma (DHG), though this entity is now increasingly recognized in young adult population as well (122). Patients with H3 G34-mutant DHG have a slightly better prognosis than H3K27M-mutant DMG, but long-term survival remains unlikely (123). It has been shown that the TME of these histone-mutant DHGs is less immunosuppressive than the histone wild-type tumors, with fewer MDSCs and increased T cell infiltration (124). It is postulated that this less suppressive TME in H3 G34-mutant tumors would allow for a more robust pro-inflammatory response in the presence of immunotherapy aimed to stimulate an antitumor response, and work in this area is ongoing.

## Development of immunotherapies for high-grade glioma

### Immune checkpoint blockade

Immune checkpoints (IC) are negative regulators of the immune system that maintain self-tolerance. These molecules are receptor-ligand pairs that following immune activation act as natural inhibitors to diminish or stop inflammation (125). However, these pathways can be activated in tumor cells, blocking immune surveillance. Thus, compounds that target IC like PD-1, PD-L1 or CTLA-4 can enhance anti-tumor immunity, allowing T cells to eliminate cancer cells more efficiently. Immune checkpoints inhibitors (ICIs) have led to increased cure rate in many aggressive solid tumors and the potential application of ICIs in HGG has been actively evaluated. Although HGGs are generally “cold tumors” due to its relatively low T cell infiltration in comparison with other tumors (126), ICI development is still may be a valid approach in gliomas, as CTLA-4, PD-1, TIM3, LAG3 and their corresponding ligands are expressed in these tumors (127).

#### CTLA-4

CTLA-4 is a co-inhibitory receptor present on the surface of APCs and Tregs (128) with the B7 family (B7-1 or CD80 and B7-2 or CD86) as their natural ligands. CTLA-4/B7 interaction inhibits T cell proliferation and cytokine production, such as IL-10, TGFβ, and indoleamine (129). CTLA-4 expression has been observed in HGG and is linked to a worse prognosis (130, 131). Preclinical studies have shown that antibodies that inhibit CTLA-4 produced antitumoral responses and promising results as single agents or in combination with other immune-stimulant approaches (132). Thus, the preclinical properties of ipilimumab and tremelimumab (fully humanized anti-CTLA-4 antibodies) led to their transition into clinical trials (133, 134). Specifically within the HGG population, a sub-analysis of the large CheckMate 143, a phase 1 clinical trial of nivolumab (anti-PD-1) alone or in combination with ipilimumab, revealed that in a recurrent GBM population, no differences in overall survival were seen with monotherapy vs. combination therapy, but higher toxicity when the anti-CTLA-4 antibody was added to the treatment (135).

#### PD-1 and PD-L1

The transmembrane receptor PD-1 (encoded by *PDCD1* gene) is a member of the immunoglobulin family that it is mainly expressed in T cells (136) and can be activated by PD-L1 (*CD274*) or PD-L2 (*CD273*), ligands known to be present in APCs, B cells and parenchymal cells (137). The PD-1/PDL1 pathway avoids autoimmunity through negative regulation of the T cell-mediated immune response (136). Several reports have shown that PD-L1 can be expressed in GBM cells (4, 138–141). In addition, it has been reported that higher levels of PD-L1 expression in GBM are correlated with worse outcome (140). Encouraging preclinical results in GBM mouse models have demonstrated that PD-L1 inhibitors could have therapeutic efficacy (142–145). However, due to the highly invasive, aggressive, and immunosuppressive phenotype of HGG, several clinical trials have shown unsatisfactory results and no survival advantage of PD-1/PD-L1 inhibitors as monotherapy (146, 147). In this regard, several clinical trials are evaluating new therapeutic approaches that combine standard-of-care therapy (temozolomide and/or radiotherapy) with molecularly-targeted therapy or immunotherapy to overcome the limited efficacy of these ICIs as monotherapy (148).

#### TIM3 and GAL9

T cell immunoglobulin and mucin domain-containing molecule 3 (TIM3) is an inhibitory receptor present in T cells that, upon activation by its ligand Galectin-9, plays a key role in abolishing T cell response against tumors (149, 150). TIM3 expression is associated with poor prognosis (59) and TMZ resistance (151) in gliomas. Moreover, it was shown that MGMT promoter methylation status in combination with TIM3 expression could be a novel prognostic signature for GBM (151). Thus, targeting TIM3 could be a promising approach for further development of immunotherapy treatments, alone and in combination with PD-1 and CTLA-4-mediated immunotherapy.

#### LAG3

Lymphocyte activation gene-3 (*LAG3* or *CD223*) is another negative regulatory molecule present on NK and T cells (152). The expression of LAG3 has been detected in 10% of gliomas biopsies in one study and in 66% of samples in other study (153). However, LAG3 was shown to be positively correlated with IDH1 expression in patients with IDH1 mutation (62). Although the expression of LAG3 is controversial in gliomas, it is possible that the expression of this immunological checkpoint could raise in response to the inflammatory infiltration induced by immunotherapeutic strategies, such as anti-mIDH vaccines. In this light, LAG3 may be an interesting target to explore further in HGG, especially in IDH1-mutant HGG.

CTLA-4 and PD-1/PD-L1 are the most studied molecules in preclinical and clinical studies (125) but at the moment, there are no FDA-approved ICIs for GBM. Thus, efforts in ICIs research for GBM should explore additional checkpoints and develop combination strategies to improve responses and expand ICIs treatments to a greater number of HGG patients. It will therefore likely be important to combine ICIs with other immunotherapeutic approaches such as those discussed below.

### Anti-cancer vaccines

The goal of cancer vaccines is to inhibit cancer progression or relapse by inducing humoral (tumor-specific antibodies) or cellular (cytotoxic T cell activation) anti-tumor responses (154). Most of the efforts to date in cancer vaccine development were focus on the latter, supported by the rationale that T cells are able to directly eliminate tumor cells in non-immunosuppressive environments.

Currently, multiple vaccine approaches are being tested in preclinical and clinical studies: peptide vaccines, DNA vaccines, cell vaccines and mRNA vaccines. Peptide or DNA vaccines involve the inoculation of tumor-specific peptides or DNA to induce a potential adaptative immune response once they reach lymph nodes. DC vaccines are derived from peripheral blood mononuclear cells (PBMCs) and are loaded with tumor antigens. Finally, mRNA vaccines encode for specific tumor antigens to elicit potent immune responses.

Peptide vaccines are composed of peptides of 8–25 amino acids in length, containing an epitope within an antigenic sequence. Because humoral responses require B cells to recognize conformational epitopes and short peptides do not emulate conformational epitopes, these vaccines are usually designed to induce cellular-mediated immunity. For this reason, peptides are design based on tumor-specific antigens (TSAs) and tumor-associated antigens (TAAs) that can be recognized by antibodies or T cell receptors (TCRs).

Proteins frequently mutated or aberrantly expressed genes in HGG include EGFR, PTEN, TERT, RB1, TP53, IDH1, PIK3CA and PIK3R1 (155). Although these pathways represent potential candidates to develop peptide vaccines, due to the high intratumor variability in expression in an individual tumor, only the targets that are consistently present in HGG have been proposed as vaccine targets. Epidermal growth factor (EGFR) is genetically altered, whether by amplification, mutation, rearrangement, or altered splicing, in almost 60% of HGG (156, 157). Constitutively active mutant EGFR has been found to promote angiogenesis through increased secretion of interleukin 8 (IL-8) and constitutive DNA binding of the transcription factor nuclear factor (NF)-kB (158). The constitutively active splice variant of epidermal growth factor (EGFR), EGFRvIII, has shown to promote tumor progression and chemoresistance in GBM, and EGFRvIII is expressed in 25–40% of GBMs (155, 159). In spite of these promising pre-clinical developments, translation to the clinic of single peptide vaccines have remained ineffective.

Thus, rindopepimut, a 14 amino acid peptide vaccine covering the EGFRvIII-specific exon junction site with keyhole limpet haemocyanin (KLH) as carrier protein was developed. Preclinical results in mice treated with rindopepimut show EGFRvIII-specific antibodies lead to antitumor immunity against EGFRvIII-positive tumor cells, inhibiting tumor growth and increasing median survival following intracerebral challenge with EGFRvIII-positive tumor cells (160). Moreover, when mice received this vaccine prior tumor implantation, tumor formation was prevented (161). A phase 1 trial (VICTORI) showed to be well-tolerated with minor adverse effects (162). Three phase 2 clinical trials followed, ACTIVATE, ACT II and ACT III, which confirmed safety and an increase in PFS and OS in vaccinated patients, compared to patients treated with TMZ (163–165). A phase 3, randomized, placebo-controlled trial (ACT IV) was designed to assess whether the addition of rindopepimut to TMZ improved survival in patients with EGFRvIII-positive GBM. However, this study did not show an improved survival in patients with newly diagnosed GBM treated with rindopepimut (166). As predicted by histological data from preclinical results, patients lost EGFRvIII expression after recurrence, proving that targeting heterogeneously expressed antigens is unlikely to be sufficient to achieve substantial clinical benefit in these patients. Taking into account these results, a clinical trial is evaluating the effect of a multi-peptide vaccine designed with several epitopes: EGFRvIII, IL-13, receptor alpha-2 (IL13Ralpha2), ephrin type A receptor 2 (EphA2), HER2/neu and YKL-40 peptides (167–170) in combination with TLR3 agonist poly-ICLC and Bevacizumab (a VEGF-blocking antibody) in recurrent GBM patients in a phase 2 trial (NCT02754362) (171).

Even though EGFRvIII has been the most widely studied target to date, other TAAs are being evaluated in GBM as potential peptide vaccines candidates. Survivin is an anti-apoptotic protein that showed upregulation in GBM in comparison with normal brain tissue and was associated with worse prognosis (172, 173). Thus, SurVaxM, a peptide mimetic survivin vaccine tested in combination with TMZ in newly diagnosed GBM, was found to be safe and produced survivin-specific CD8+ T cells and antibodies in a phase 2 clinical trial (174). Moreover, treatment with SurVaxM improved overall survival at 12 months (OS12) in patients with poor prognostic factors (unmethylated MGMT, higher survivin levels).

IDH, as discussed above, is a target of interest in HGG, as it is mutated in a sizeable subset of HGG and represents a protein product that is mutated only in tumor cells but not in healthy brain tissue (62). Antitumor efficacy of R123H-IDH1 peptide vaccines was assessed in transgenic MHC-humanized mice harboring mIDH1 gliomas (175). This vaccine mimics the specific mutation present in 95% of the patients with mIDH1 (176) and it has been shown to induce antitumor immune responses that correlate with increased survival of mice with orthotropic gliomas (177). Thus, the potential success of peptide vaccines targeting mIDH1 is being evaluated in mIDH1-glioma patients (NOA-16 in NCT02454634, PEPIDH1M in NCT02193347) (178). Promising combination strategies were recently published and reviewed in detail (179–181).

Dendritic cells (DCs) are antigen-presenting cells that recognize, process and present antigens to T cells promoting an adaptive anti-tumor immune response (181). Approaches to use DCs in anti-tumor vaccines have been developed, where DCs are loaded with TAA, peptides, viral antigens, RNA, or tumor lysates are administered and subsequently lead to an antitumoral T cell response and tumor cell lysis and prevent tumor recurrence (182). DCs vaccination for GBM has been tested in several preclinical mouse models (183–185), prophylactic (183, 186, 187), and therapeutic (188–194) settings, and have been shown to be safe, without inducing autoimmunity, leading to reduced tumor growth and prolonged survival. Thus, although clinical efficacy of DCs vaccination is not robust at this time, these animal studies provided strong rationale for the continued optimization of DCs vaccine development and clinical development in patients with HGG (195).

### CAR T cells

**Figure 1:**
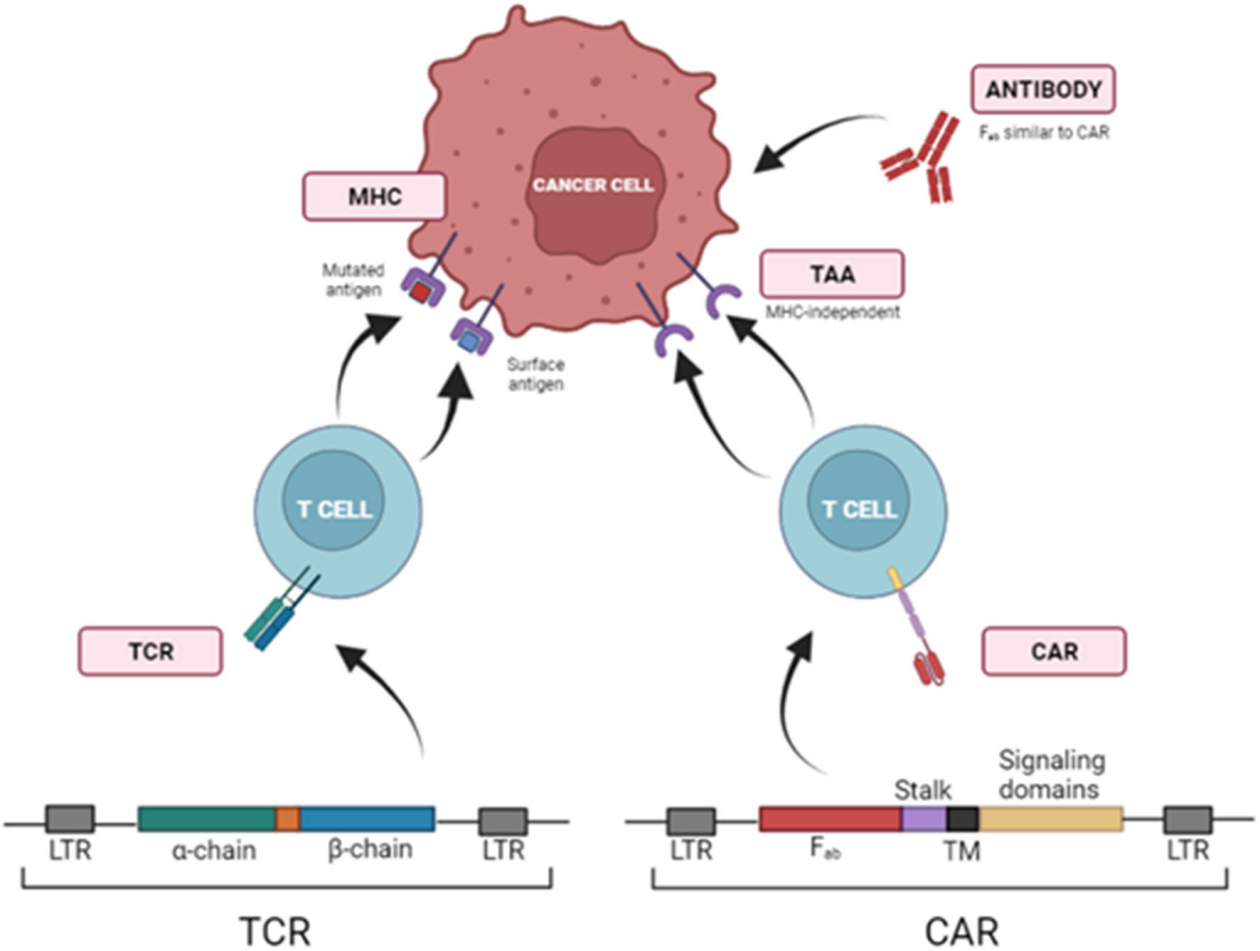
Strategies to redirect T cells toward tumor cells using TCRs and CARs. T cells can be redirected against TAAs *via* viral transduction of specific T Cell Receptors (TCRs) or Chimeric Antigen Receptors (CARs), which identify target molecules in the surface of tumor cells independently of MHCI presentation. LTR, Long Terminal Repeat; Fab, fragment antigen-binding region; TM, transmembrane domain.

T cells can be modified to be redirected against TAA *via* viral transduction of T Cell Receptors (TCRs) or Chimeric Antigen Receptors (CARs). Transferring TCR chains is limited to antigens presented in the context of MHC or human leukocyte antigen (HLA). To avoid this requisite, T cells can be redirected to TAA through the transduction of chimeric antigen receptors (CARs) (Figure 1).

Chimeric antigen receptors (CARs) are recombinant receptors that combine the effector functions of T cells and the ability of antibodies to recognize specific targets found on cancer cells in a non-MHC restricted manner. CARs generally consist of an extracellular antigen-recognition domain (typically an antibody single-chain variable fragment (scFv), but it can be a peptide or other protein). This domain is linked to an intracellular signaling domain. CARs' design has started with first-generation CARs that included the CD3ζ of TCR. Subsequent CAR development (second, third and fourth generations) added other intracellular domains from CD28, 4-1BB, or OX40 to CD3ζ, trying to emulate co-stimulation signals required for a complete T cell activation (196).

The first clinical trials of CAR T cvells enrolled patients with CD19-positive hematological cancers, such as B-cell acute lymphoblastic leukemia (B-ALL) (197–199) or chronic lymphocytic leukemia (CLL) (200–203). Although the initial CAR T cell clinical trials were conducted in patients with advanced blood cancers and barriers in the use of CAR T cells in solid tumors, there remain a large number of preclinical and clinical studies aimed at optimizing this treatment in patients with solid tumors. As discussed, the generally immunosuppressive TME and other biological features of HGG present challenges to the successful development of cytotoxic or targeted chemotherapies. In this sense, CAR T cell therapy holds significant promise as an emerging strategy in the treatment of aggressive solid tumors such as HGG.

Several GBM antigens represent reasonable targets for CAR T cell development, such as EGFRvIII (204, 205), IL13 receptor subunit alpha 2 (IL13Ra2) (96, 206, 207) or Her2 (208–210). However, although CAR T cell development has shown promise, one of the most important limitations of its application in HGG is the heterogeneity of these tumors. This heterogeneity makes it difficult to develop CAR T cell-based strategies to target all clonal and subclonal populations (211). In this regard, CAR modifications are being developed to diminish tumor antigen escape and overcome the heterogeneity and immunosuppressive TME in HGG. These novel approaches can give CAR T cells the ability to produce epitope spreading, stimulating tumor-specific immune responses.

One of the first attempts to enhance this approach was to modify T cells with a bicistronic retroviral vector encoding the CD40 ligand (CD40L) gene in addition to the CD19-specific CAR. The expression of the immune-stimulatory molecule showed tumor specific cytotoxic effects in different mouse models of CD40^+^-CD19^+^ B-cells and patient-derived chronic lymphocytic leukemia cells (212). Moreover, these CD40L-CAR T cells prolonged overall survival of CD19+ tumor-bearing mice when compared to CD19-CAR T cell treatment (212). In another study, CD40L-CAR T cells displayed greater antitumor effects, recruiting enhanced levels of immune effectors and inducing a sustained antigen-presenting cell response to mobilize antitumor T cells (213). Moreover, these CAR T cells were capable of orchestrating an endogenous T cell-mediated response against unidentified tumor antigens (213). Another study explored the potent antitumor effects of the CD40 pathway by modifying CAR T cell to secrete anti-CD40 agonist antibodies (214). These cells not only secreted increased levels of cytokines, but these treatments also led to an enhanced antitumor activity *in vivo* in a human ovarian cancer model (214).

As mentioned above, using CAR T cells for producing a host immune response against unknown tumor antigens may be an interesting strategy to overcome an at-present somewhat limited efficacy of CAR T cells in the treatment of HGG. In this regard, engineered T cells that express and secrete Flt3L, a DC growth factor, were developed for the treatment of solid tumors (215). Flt3L-secreting CAR T cells not only promoted DC expansion and differentiation, but also enhanced DC-dependent and T cell-mediated inhibition of tumor growth by inducing endogenous epitope spreading.

Chemokine receptors present in the tumor microenvironment (TME) are capable of recruiting different subsets of leukocytes into tumors which share similar patterns of chemokine expression. This feature raises the possibility of modifying CAR-T cells to enhance its infiltration into solid tumors using key chemokine-chemokine receptor axes. MDSCs are recruited *via* CXCR1/CXCR2/CXCR4/CCR2 (216) and it was shown that CXCL12 expression, is upregulated in cancer-associated fibroblasts in many solid tumors (216–218). Moreover, the CXCR4-CXCL12 axis has been associated with higher proliferation rates, angiogenesis, and metastasis of several cancers (219). To enhance the recruitment of CAR T cells into the bone marrow of mice transplanted with a patient-derived acute myeloid leukemia (AML), CD25 targeted CAR T cells were modified to also express CXCR4 (220). The injection of these CXCR4-modified CAR T cells resulted in complete remission of human AML cells in peripheral blood diminishing tumor burden (220). Moreover, CXCR4-modified CAR T cells that target B cell maturation antigen (BCMA) are being tested in a phase 1 study in patients with multiple myeloma (NCT04727008).

Although these next-generation CAR T cells have shown promising results in hematological cancers, the major challenge to use them in solid tumors is the dense in anti-inflammatory TME that prevents effective homing of the therapy. Thus, the ability of these new CARs to produce epitope spreading or the production of chemokine receptor-modified CAR-T cells, may enhance their efficacy against solid tumors, with the aim of engaging as many immune effectors as possible and produce a more potent antitumor effect.

### Gene therapy

Gene therapy is a therapeutic approach that consists of manipulating genetic elements to treat diseases such as glioma. In this approach, complete genes, oligonucleotides, or different regulatory elements may be delivered into the target glioma cells either by mechanical methods or by using vectors. To achieve high therapeutic efficacy, vectors must be chosen, considering the expression levels of therapeutic transgene, immunogenicity, biosafety and distribution of gene expression within the TME (221–223). The advantage of using gene therapy is its local administration may overcome the challenges exerted by the BBB for systemic delivery. Several viral and non-viral immune stimulatory gene therapies have shown efficacy in many pre-clinical studies; however, their successful clinical implementation still manifests several challenges (224).

Immune stimulatory gene therapy involves tumor-selective gene transfer of various cytokines such as IL12 and IFNs which can induce robust immune responses in glioma cells (222). Gliomas can effectively evade host immune responses (225). In this respect, in a phase 2 clinical trial GBM patients with unmethylated MGMT promoter were administered with a single dose of autologous CD34+-enriched hematopoietic stem and progenitor cells (HSPCs) exposed to transduction with a 3rd generation lentivirus mediating myeloid-specific IFN-α2 expression (NCT03866109). In another study, non-replicative adeno-associated virus (AAV) and replicative Herpes simplex virus (HSV) have been used to express IL12 in malignant glioma, resulting in significant inhibition of tumor and increased expression of IFNγ together with microglial activation and recruitment of T and NK-cells (226, 227). Recently, two phase 1 clinical trials (NCT02026271 and NCT03330197) revealed that when the resection cavity walls were injected with a fixed dose of a regulatable Ad-RTS (RheoSwitch Therapeutic System)-hIL12 vector along with veledimex, an oral activator of IL-12, increased expression of IFNγ in peripheral blood of the glioma patients was seen (228). Additionally, an increase in TILs and PD1+ immune population was observed following Ad-RTS-hIL12 treatment (228). These inflammatory infiltrates support the immunological anti-glioma effect of IL-12 and IFNs through gene therapy.

**Figure 2:**
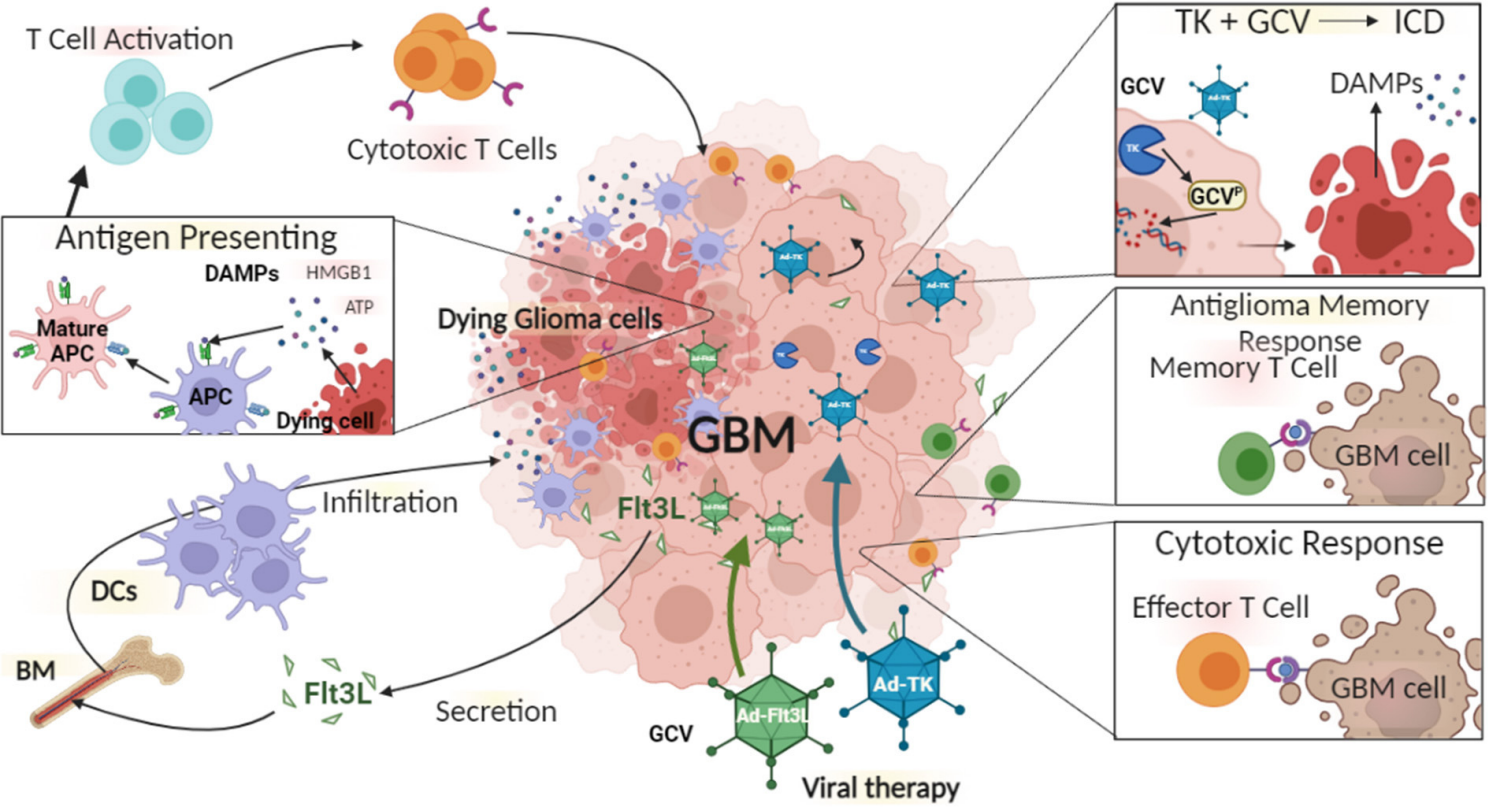
The immune mediated gene therapy consisting of Ad-TK [plus Ganciclovir (GCV)], and Ad-Flt3L. Glioma cells can be efficiently transduced with Ad-TK, which encodes the conditionally cytotoxic HSV1-Thymidine Kinase (TK). TK can convert the prodrug GCV, a nucleotide analog, to GCV-phosphate, which is further phosphorilated into GCV-triphosphate by intracellular kinases. GCV-triphosphate is a purine analog and can inhibits DNA replication, inducing Immunogenic Cell Death (ICD). Dying cells release Damage-Associated Molecular Patterns (DAMPs), i.e., HMBG1, calreticulin, and ATP. Glioma cells transduced with Ad-Flt3L, express and secret Flt3L that recruits dendritic cells (DC) into the tumor microenvironment, where they uptake tumor associated antigens and get activated by DAMPs. Mature antigen presenting cells (APC) migrate to the regional lymph nodes and prime anti-tumor cytotoxic CD8+ T lymphocytes. Cytotoxic T cells recognize and kill tumor cells. Following T cells exposure to tumor antigen, immunological memory is developed. Memory T cells activate an anti-tumor response leading to inhibition of tumor recurrence.

With the aim of overcoming the limitations of monotherapies, combination therapies together with gene therapy have been developed. We have pioneered the combination of Ad-Flt3L and Ad-TK for glioma therapy. The expression of HSV1-TK within glioma cells in the presence of systemic delivery of ganciclovir (GCV) leads to DNA replication termination, which ultimately results in glioma cell death (Figure 2) (223, 229). This induces the release of tumor antigens and damage-associated molecular patterns (DAMPs) molecules into the TME and triggers immune responses *via* the activation of DCs and generation of T cell mediated cytotoxic immune responses against glioma-antigens (230). These infiltrating DCs can phagocytose antigens that are released from TK-induced glioma cell death (223). Also, Flt3L increases the migration and infiltration of DCs into the TME (223). We found that combination therapy resulted in long-term survival of glioma-bearing animals compared to either therapy used and monotherapy (8). Also, our combination gene therapy together with CTLA4, or anti-PD-L1 immune-checkpoint blockade significantly increased the survival of glioma-bearing animals (8). Our first human phase 1 trial (NCT01811992) using Ad-Flt3L and HSV1-TK combination gene therapy for the treatment of newly-diagnosed, resectable malignant gliomas, revealed increased levels of DCs, CD4+ and CD8+ T cells within the TME (231). Therefore, our results showed for the first time that reprogramming the host's brain immune system to recognize glioma antigens could present an attractive approach to the treatment of glioma (224).

### Oncolytic viruses

The use of oncolytic virotherapy represents an attractive therapeutic approach for the treatment of HGG. Oncolytic viruses (OVs) are viruses that selectively infect and lyse cancer cells and spare normal surrounding cells. OVs are designed to recognize tumor-specific receptors or to replicate under oncogene promoters to improve their tropism and avoid non-malignant cells. It was observed that an immunosuppressive TME promotes the infectivity of oncolytic viruses and improves oncolysis (232, 233). Once the tumor cells are infected with OVs, the dying tumor cells present tumor-associated antigens/epitopes that are released in the TME and trigger a viral or tumor-specific T cell-mediated immune responses, critical for the efficacy of oncolytic virotherapy and overcoming tumor-mediated immunosuppression (234, 235).

HSV G47Δ-mIL12, a genetically engineered third-generation oncolytic virus armed with IL-12, showed increased survival in a syngeneic murine GBM model (236). Recently, HSV G47Δ was evaluated in a phase 2 clinical trial in patients with GBM who received repeated intratumoral stereotactic injections in addition to TMZ (237). Vectors based on Newcastle disease viruses (NDV) have a natural tropism for tumor cells and also have oncolytic potential and immune-stimulatory properties (238). LaSota strain of naturally oncolytic NDV can induce increased apoptosis in glioma when treated complementarily in comparison to TMZ alone, and combination treatment significantly enhances survival in a rat xenograft tumor model (239). Moreover, *in vivo* immune-virotherapy with stains of measles virus (MV) in combination with anti-PD-L1 blockade synergistically enhanced the infiltration of activated CD8+ T cells within the TME and increased the survival of the syngeneic GBM mouse model (240). Recently, MV has been evaluated in a dose-finding phase 1 clinical trial in patients with recurrent HGG, and no dose-limiting toxicities were observed (NCT00390299). In addition, an oncolytic double-stranded human RNA *orthoreovirus* (known as reovirus) is under clinical trial as Reolysin in patients with GBM (NCT00528684) (241). Reovirus can selectively target and lyse Ras-activated malignant cells (242). Currently, a phase 1 clinical trial is evaluating the combination of intravenously administered Reolysin and subcutaneously administered GM-CSF on patients with recurrent HGG (NCT02444546). Another phase 2 trial with conditionally replicating oncolytic-HSV1 viz. G207 revealed anti-tumor activity without any serious adverse effects (NCT00028158). Recently reported results from a phase 2 trial in Japan (UMIN000015995) employing HSV G47Δ, showed a survival benefit and good safety profile in patients with residual or recurrent GBM. Patients received repeated intratumoral stereotactic injections for up to six doses. Overall median survival was 20.2 (16.8–23.6) months after G47Δ initiation and 28.8 (20.1–37.5) months from the initial surgery (243). In contrast, recurrent glioblastoma reported median survival after standard of care treatment is ~5.0 months (244). Gene therapy employing oncolytic viruses represents a promising treatment for GBM, although OVs may still require additional engineering to generate an OV that persists and spreads effectively, while being massively lytic for tumor cells.

## Challenges in immunotherapy development for high-grade glioma

Although immunotherapy for the treatment of CNS tumors is an area of active research, several barriers to the development of successful immunotherapies remain. Significant intra- and intertumoral heterogeneity occurs in both adult and pediatric HGG and the genetic and epigenetic drivers of tumorigenesis can evolve over time as tumors progress (120, 245–247). A deeper characterization of the variations in immune infiltrates within the TME of HGG is critical to understand how immunotherapy can be used to induce an antitumor response, possibly most effectively through a multi-modal therapeutic approach using immunotherapy combined with targeted therapies. Successful delivery of an immunotherapy to the tumor site is critical to testing its efficacy rigorously. The blood-brain barrier (BBB) has impaired successful transition of therapies developed for non-CNS solid tumors into CNS tumors (248). However, local delivery of immunotherapy mitigates this problem and either direct injection of a therapy intraoperatively or administration *via* an Ommaya reservoir, for example, are approaches currently being used in immunotherapy clinical trials for CNS tumors (5). The field of immunotherapy in the treatment of cancer has only relatively recently achieved significant successes in certain tumor types (249, 250), and there is much that remains to be learned regarding how to better translate preclinical immunotherapeutic advances into the clinic, as there are often many unknowns and practical challenges that arise when immunotherapies are transitioned into first-in-human studies. Collaborative, “team-science” will be increasingly necessary to efficiently and effectively develop successful immunotherapies for HGG.

### Therapy development in adult vs. pediatric high-grade glioma

An advance in the treatment of HGG, specifically GBM, was made in 2005 after completion of a randomized phase 3 study of radiotherapy (RT) alone vs. RT plus concurrent temozolomide, where median survival improved from 12.1 months with RT alone to 14.6 months with RT plus TMZ (251). Additionally, analysis of several clinical trials *via* meta-analysis revealed the addition of bevacizumab to RT led to improved progression-free survival (PFS), but not OS or overall survival at 6 months (OS6) (251, 252). A multitude of similar chemotherapy regimens have subsequently been trialed in pediatric populations, and unfortunately a parallel survival benefit has not been seen with identical regimens or other combinatorial cytotoxic chemotherapeutic regimens (253–256). The differential benefit, or lack thereof, derived from these therapeutic approaches between adult and pediatric patients with HGG is thought to be primarily the result of disparate tumor biology between these two age populations. The genetic changes and activated pathways in each HGG population were rather incompletely understood at the time these trials were implemented, where tumor histology was considered the predictor of biology and clinical behavior. Therefore, clinical trials that used only a histological diagnosis for study entry with a lack of tumor-specific genetic data are now understood to have enrolled a rather heterogenous group of patients. This has made extrapolation of results from the adult HGG population to a pediatric population much less accurate in predicting response. As a result, there is an increasingly strong sentiment in the pediatric oncology community that the tumor biology of pediatric HGG, and the multiple molecularly distinct entities within this diagnosis, should drive specific therapeutic development as opposed to transition of therapies from adult HGG into pediatric patients with HGG (257). Unfortunately, although we have a much deeper insight into the genetic and epigenetic landscape of HGG in both pediatric and adult populations, the treatment paradigm of surgical resection and radiotherapy +/- alkylating chemotherapy remains unchanged with rather negligible improvement in clinical outcomes.

These prior failures of therapies to successfully translate into benefit in a pediatric population after one was seen in adult patients are likely multifactorial, but it has become increasingly clear that the developmentally linked genetic and epigenetic alterations found in pediatric cancers in general, and pHGG specifically, lead to significant differences in tumor biology. Future therapy successes in pHGG may be those transitioned from adult therapeutic successes. However, entirely different approaches according to the tumor biology may be required to successfully develop immunotherapies and targeted therapies for the treatment of pHGG.

### Practical challenges in CNS immunotherapy clinical trials

There are several clinical trial related barriers that can prove challenging in the development of immunotherapy for HGG. Cell-based and gene-based immunotherapies require a large infrastructure for cell or vector manufacturing with multiple layers of regulatory oversight necessary to maintain product safety. Although this is true for clinical trials in general, immunotherapies and the clinical infrastructure necessary to deliver these immunotherapies are expensive and require significant support beyond what is necessary to complete trials with generic cytotoxic or targeted chemotherapy.

If possible, CNS immunotherapy studies should be expanded beyond single institution studies into multiple institutions preferably under the umbrella of a consortium, as consortia-based trials increase patient access, support robust accrual to these clinical trials, and allow for broader access to a novel therapy that is not as limited by patient geography. Another important factor in the design of immunotherapy clinical trials is the incorporation of appropriate biomarkers into the studies. Ideally, the chosen biomarkers should have either been validated or supported by strong preclinical data instead of more broadly drawing patient blood during a trial and deciding after-the-fact the testing that will be done. Careful consideration of the design and incorporation of biomarker studies into a clinical trial testing an immunotherapy is critical to make the most of precious, and often scarce for practical reasons, samples obtained from patients who agree to enroll on these studies.

## Conclusions

Novel therapeutic approaches for glioma have been developed, and although they showed promising results in preclinical models, they ultimately failed in Phase 3 clinical trials. Some of the obstacles that may hamper therapeutic efficacy in the clinic include the highly heterogeneous nature of gliomas, the presence of the BBB- that precludes the entry of drugs to the CNS-, tumor immune escape, invasion of glioma cells into the surrounding brain tissue, and the immune suppressive nature of the glioma microenvironment.

Preclinical models currently available to assess the efficacy of immune-mediated therapies, include, tumor syngeneic models established in immunocompetent rodents. Whilst these models are useful to study the cross talk between the tumor cells and the immune microenvironment, they fail to replicate tumor heterogeneity as encountered in human patients. Thus, although single tumor antigens used as vaccine targets have yielded encouraging results in these models, they failed in Phase 3 clinical trials (163, 166). Recently, more accurate heterogeneous tumor models have encompassed the generation of genetically engineered mouse models of glioma (GEMMs) and have been implemented in immunocompetent rodents (72, 258–260). These models more accurately recapitulate the salient mutations encountered in different glioma subtypes and harbor tumor heterogeneity. Thus, they constitute attractive preclinical models to test the efficacy of immunotherapies. An additional challenge related to the available glioma models is the small tumor size achievable on rodent models. However, to overcome this, pet dogs which exhibit endogenous gliomas are an attractive model, as the tumors harbor many features encountered in glioma human patients, including tumor heterogeneity, an immunosuppressive TME and the presence of the BBB (261–263).

Many promising immune-mediated therapies have been implemented in the clinic with disappointing outcomes, these include immune checkpoint blockade, CAR T cells, peptide vaccines, dendritic cells-mediated vaccines, gene therapies and oncolytic virotherapy. To date, these therapies have been implemented as monotherapies, perhaps, the key would be to use them in combination to maximize efficacy. In this regard, one could envisage combining immune checkpoint blockage with immune stimulatory gene therapy to prevent T cell exhaustion and maximize the activity of cytotoxic T cells within the TME. This will in turn enhance the efficacy of CD8 T cells mediated glioma cells' killing.

In summary, HGG patients have a poor prognosis and effective therapies are not available. Currently, the standard of care for HGG involves maximal safe surgical resection, radiation therapy, and treatment with temozolomide. Despite advances in surgical and imaging techniques, the median survival for these patients has improved only marginally over the past decades. This highlights the need to expand our translational research efforts to improve outcomes for these patients.

Upcoming scientific discoveries will further reveal mechanisms which mediate immunosuppression and cross talk between glioma cells, brain resident cells and immune cells within the TME, providing appealing signaling pathways for developing novel therapeutic strategies. In addition, with the advent of scRNA-seq technologies and its application to uncover mechanisms of resistance, powerful targeted therapies may emerge to prevent tumor recurrence. Moreover, targeting known mutations, such as in mutant IDH, represent exciting avenues for developing novel immunotherapies harnessing epigenetic manipulations of the tumor immune microenvironment (4, 54). Novel translational research followed by the implementation of clinical trials to assess the efficacy and safety of these exciting therapies, should lead to improved median survival for these patients.

## Author contributions

AF, BM, MV, AC, MS, KB, ZZ, NG, and MC wrote the manuscript with overall guidance, revisions, and edits from PL and MGC. All authors read and approved the final version of the manuscript.

## Funding

This work was supported by the National Institutes of Health/National Institute of Neurological Disorders and Stroke (NIH/NINDS) Grants R37-NS094804, R01-NS105556, R01-NS122536, R01-NS124167, and R21-NS123879-01 and Rogel Cancer Center Scholar Award to MGC; NIH/NINDS Grants R01-NS076991, R01-NS082311, R01-NS096756, and R01NS122234; and NIH/NCI R01-CA243916 to PL; the Department of Neurosurgery; the Pediatric Brain Tumor Foundation (PBTF), Leah's Happy Hearts Foundation, Ian's Friends Foundation (IFF), Chad Tough Foundation, and Smiles for Sophie Forever Foundation to MGC and PL; Rogel Cancer Center Clinical Research Early Investigator Award, the Department of Pediatrics, the Chad Carr Pediatric Brain Tumor Center to AF; NCI CCSG Grant P30CA046592 to MGC, PL, and AF; Agencia Nacional de Promoción Científica y Tecnológica (PICT 2018–3088 and PICT 2019-00117 to MC and post-doctoral fellowship to NG).

## Conflict of interest

The authors declare that the research was conducted in the absence of any commercial or financial relationships that could be construed as a potential conflict of interest.

## Publisher's note

All claims expressed in this article are solely those of the authors and do not necessarily represent those of their affiliated organizations, or those of the publisher, the editors and the reviewers. Any product that may be evaluated in this article, or claim that may be made by its manufacturer, is not guaranteed or endorsed by the publisher.
